# Exosomal miR-92b-5p regulates N4BP1 to enhance PTEN mono-ubiquitination in doxorubicin-resistant AML

**DOI:** 10.20517/cdr.2024.140

**Published:** 2025-03-28

**Authors:** Qianyuan Li, Jie Cheng, Danni Qin, Sheng Xiao, Chenjiao Yao

**Affiliations:** ^1^Department of General Medicine, The 3rd Xiangya Hospital, Central South University, Changsha 410013, Hunan, China.; ^2^Department of Hematology, The 3rd Xiangya Hospital, Central South University, Changsha 410013, Hunan, China.; ^3^Department of Pathology, The 3rd Xiangya Hospital, Central South University, Changsha 410013, Hunan, China.; ^4^Department of Hematology, The First Affiliated Hospital of Hainan Medical University, Haikou 570105, Hainan, China.

**Keywords:** Exosomal miR-92b-5p, N4BP1, NEDD4, PTEN, doxorubicin resistance, AML

## Abstract

**Aim:** Doxorubicin, pivotal for acute myeloid leukemia (AML) treatment, often succumbs to resistance, impeding therapeutic success. Although exosomal transfer is linked to chemoresistance, the detailed role of exosomal miRNAs in doxorubicin resistance remains incompletely understood.

**Methods:** We employed miRNA sequencing to delineate the profile of exosomal miRNAs in doxorubicin-resistant K562/DOX cells and AML patients. Subsequently, qPCR was utilized to scrutinize the expression of exosomal miR-92b-5p in these resistant cells and AML patients. A dual-luciferase reporter assay was conducted to elucidate the direct binding of miR-92b-5p to NEDD4 binding protein 1 (N4BP1). Furthermore, interactions between N4BP1 and NEDD4, as well as between NEDD4 and PTEN, were investigated by co-immunoprecipitation (Co-IP). Meanwhile, the ubiquitination of PTEN was also examined by Co-IP. Western blot analysis was applied to assess the expression levels of N4BP1, NEDD4, PTEN, RAD51, and proteins associated with the PI3K-AKT-mTOR pathway. Gain- and loss-of-function studies were conducted to ascertain the functional role of miR-92b-5p in doxorubicin resistance by using miR-92b-5p-mimic and miR-92b-5p-inhibitor transfections.

**Results:** Our study found exosomal miR-92b-5p was upregulated both in doxorubicin-resistant cells and AML patients. Moreover, miR-92b-5p targets N4BP1, promoting NEDD4-mediated mono-ubiquitination of PTEN. This alters PTEN’s subcellular localization, promoting nuclear PTEN and reducing cytoplasmic PTEN, which in turn leads to increased RAD51 for DNA repair and activation of the PI3K-AKT-mTOR pathway for cell proliferation, contributing to doxorubicin resistance.

**Conclusion:** Our study reveals a novel mechanism of doxorubicin resistance mediated by exosomal miR-92b-5p and provides potential therapeutic targets for overcoming drug resistance in AML.

## INTRODUCTION

Acute myeloid leukemia (AML) is a hematological malignancy marked by the proliferation of abnormal white blood cells, severely impacting blood production. Doxorubicin, a widely utilized anthracycline antibiotic, is a cornerstone of AML chemotherapy due to its ability to intercalate DNA and generate free radicals, thereby inhibiting DNA replication and transcription. Despite advances in chemotherapeutic regimens, the emergence of doxorubicin resistance remains a significant challenge, undermining treatment efficacy and leading to poor patient outcomes^[1]^. There is a critical need to understand the molecular underpinnings of this resistance to develop novel therapeutic strategies.

MicroRNAs (miRNAs), which are 18-24 nucleotide non-coding RNAs, interact with the miRNA-induced silencing complex (miRISC) to target and regulate the expression of genes involved in the cell cycle, proliferation, apoptosis, DNA damage repair, and drug metabolism, thereby influencing drug resistance in leukemia^[2-4]^. For example, miR-128 overexpression led to increased DNA damage, which subsequently reduced cell viability and enhanced the cells’ sensitivity to etoposide and doxorubicin^[5]^. Additionally, miR-133 has been reported to boost doxorubicin sensitivity in Evi1-overexpressing cells by promoting apoptosis but has no effect in Evi1-deficient cells^[6]^. Furthermore, miR-130a knockdown increased the sensitivity of AML cells to doxorubicin by decreasing cell viability^[7]^. Nevertheless, there remains a subset of AML patients with doxorubicin resistance that cannot be attributed to the known miRNAs and their mechanisms. Therefore, it is imperative to discover novel miRNAs and explore their underlying mechanisms, which could provide fresh insights for the diagnosis, treatment, and prognostic evaluation of AML.

Exosomes are tiny vesicles released by cells and packed with miRNAs and other small molecular cargo^[8]^. Exosomes facilitate the transfer of miRNAs between cells, thereby fostering cancer progression and drug resistance by altering drug targets, reducing intracellular drug concentrations, and regulating cell cycle, apoptosis, and DNA damage repair in recipient cells^[9-14]^. For example, exosomal miR-365 from imatinib-resistant chronic myeloid leukemia (CML) K562/G01 cells confer imatinib resistance to sensitive K562 cells by inhibiting Bax and caspase-3^[15]^. It has been reported that exosomal miR-19b and miR-20a in multidrug-resistant AML HL-60/ADR cells spread multidrug resistance among sensitive HL-60 cells upon co-culture^[16]^. Despite these findings, there is a notable gap in research specifically investigating the role of exosomal miRNAs in doxorubicin resistance in AML patients and cell lines. Therefore, this study mainly investigated the exosomal miRNA in doxorubicin-resistant AML, explored and verified its target genes and pathways, thereby providing new targets and ideas for the treatment of doxorubicin-resistant AML.

## METHODS

### Study participants

Blood samples were collected from AML patients before the initiation of chemotherapy. The patient cohort comprised 5 patients with newly diagnosed AML who responded to chemotherapy and 5 patients with refractory AML who did not respond to the doxorubicin-included regimen between January 2022 and December 2023 [Supplementary Table 1]. The diagnosis and relapse/refractory status of AML were proved by morphology examination, flow cytometry analysis, karyotype analysis, and molecular detection.

### Cell culture and transfection

Human leukemia cell K562 and its doxorubicin-resistant cell line K562/DOX were obtained from the Cell Center of Xiangya Medical College and were authenticated by STR profiling (Genetic Testing Biotechnology, Suzhou, China). K562 cells were cultured in RPMI-1640 culture medium (Gibco, USA) with 10% FBS (11012-8611, EVERYGREEN, Zhejiang, China), K562/DOX cells were cultured with 1 μM doxorubicin (KGA8184, KeyGen, Jiangsu, China) additionally for maintaining resistance and were cultured in doxorubicin-free culture medium before experiment.

After cell confluence reached 70%-80%, K562 or K562/DOX cells (1 × 10^6^ cells/well) were inoculated into 6-well plates and transfected with 100 nM miR-92b-5p mimic or inhibitor (R10034.9, RIBO, Guangzhou, China) for 6 h and cultured for 48 h.

### Exosome isolation and identification

After cell confluence reached 70%-80%, K562 or K562/DOX cells were washed twice with PBS (G4202, Servicebio, Wuhan, China) and cultured in RPMI-1640 culture medium with 5% exosome-depleted FBS (Cat#EXO-FBS-50A-1, SBI, Shanghai, China) for 24 h. The cell suspensions were centrifuged at 300 *g* for 10 min at 4 °C. The supernatants were collected and centrifuged at 3,000 *g* for 10 min at 4 °C, then filtered with a 0.22 µm filter, and finally concentrated using 100 kD ultrafiltration tube (Merck Millipore, Germany). Exosomes were isolated using Exosome Purification Kit-Exosupur (Cat# Echo9101A-30ml, Echo Biotech, Beijing, China) following the manufacturer’s protocols.

The patient’s blood samples were collected with an EDTA anticoagulant tube and centrifuged at 1,500 *g* for 20 min at 4 °C. The supernatants were collected and centrifuged at 3,000 *g* for 15 min at 4 °C. Exosomes were isolated using Exosome Purification Kit-Exosupur (Cat#Echo9101A-10ml, Echo Biotech, Beijing, China) following the manufacturer’s protocols.

Exosome nanoparticle size distribution was confirmed by Nanoparticle Tracking Analysis [Supplementary Figure 1A]. The morphology of the exosome was identified by transmission electron microscopy (TEM) at the Institute of Pathology, Xiangya Medical College of Central South University [Supplementary Figure 1B]. The exosome markers calnexin, Hsp90, CD63, and Alix were detected by Western blots [Supplementary Figure 1C].

### Small RNA extraction and sequencing

Small RNA was extracted using the miRNeasy Mini Kit (Cat. No. 217004, Qiagen, Germany) following the manufacturer’s protocols. Subsequently, small RNA libraries were constructed and sequenced on Illumina HiSeq2500 platforms by Echo Biotech company (Beijing, China). MiRNA libraries were constructed using QsRNA-seq^[17]^, and unique molecular identifiers (UMIs) were employed to avoid bias during PCR. The miRNA expression levels were normalized using the TPM algorithm.

### Doxorubicin resistance-related miRNAs and target gene functional annotation

Differential expression analysis of two groups was performed using the edgeR R package (3.12.1)^[18]^. |log2(FoldChange)| ≥ 0.584962500721156; *P* value ≤ 0.05 found by edgeR were assigned as differentially expressed. To ensure the biological relevance of our analysis, we have filtered out miRNAs with low expression levels. Specifically, we have retained only those miRNAs with an average expression level (TPM) greater than 10 for our functional annotation analysis.

MiRanda^[19]^ and RNAhybrid^[20]^ were used for miRNAs target gene prediction, and BLAST software was used to compare the predicted target gene sequence with databases such as NR^[21]^, Swiss Prot^[22]^, gene ontology (GO)^[23]^, COG^[24]^, Kyoto Encyclopedia of Genes and Genomes (KEGG)^[25]^, KOG^[26]^, and Pfam^[27]^ for the miRNA target gene functional annotation. The topGO R package was utilized to perform GO enrichment analysis on the target genes of differentially expressed miRNAs. KOBAS^[28]^ was employed to assess the statistical enrichment of differentially expressed miRNA target genes in KEGG pathways.

### q-PCR

The Pubmed GENE database (https://pubmed.ncbi.nlm.nih.gov) and miRBase database (https://mirbase.org) were used for the sequence of the mRNAs and miR-92b-5p and primer sequences presented in Supplementary Table 2 were synthesized by Tsingke Biotechnology (Beijing, China). Complementary DNA (cDNA) was synthesized using TransScript ® miRNA First-Strand cDNA Synthesis SuperMix (AT351, TransGen, Beijing, China) for miRNAs and HiScript ® II Q RT SuperMix for qPCR (R223, Vazyme, Nanjing China) for mRNAs. The relative expression of miR-92b-5p, and U6, PTEN, NEDD4 binding protein 1 (N4BP1), and GAPDH mRNA was measured with PerfectStart® Green qPCR SuperMix (AQ601, TransGen, Beijing, China) on a LightCycler 480 II Real-Time PCR System (Roche, Germany) according to the instructions. U6 was used as the endogenous control for miRNAs, while GAPDH was used as the endogenous control for mRNAs, and relative expression levels were assessed with the 2^-ΔΔCT^ method.

### Protein extraction and western blots

The cells were treated with the RIPA buffer (KGP702, KeyGen, Jiangsu, China) to obtain total protein, and the nuclear and cytoplasmic proteins were extracted using a cytoplasmic and nuclear protein extraction kit (KGP150, KeyGen, Jiangsu, China). The protein was loaded onto a 10% SDS-PAGE gel and subsequently transferred to a PVDF membrane. The membrane was blocked with 5% BSA in TBST and incubated with primary antibodies overnight at 4 °C. Afterward, HRP-linked anti-rabbit IgG secondary antibody (BL003A, Biosharp, Beijing, China) was applied at a 1:10,000 dilution and incubated at 25 °C for 1 h. The protein bands were visualized using ECL reagents (BL520A, biosharp, Beijing, China). The Tublin, PCNA, and GAPDH protein levels were used as internal controls. Primary antibodies are listed in Supplementary Table 3.

### Dual-luciferase assay

We purchased psiCHECK2 vectors with N4BP1 3’-UTRs wild-type (WT) or mutant (MUT) sequences from Changsha Abiowell Biotechnology (Changsha, China). 293T cells were transfected with one of the above vectors along with the miR-92b-5p mimic or mimic-NC using Lipofectamine 2000 (Thermo Fisher Scientific, USA) for 72 h. Luciferase activities were measured using the TransDetect Double-Luciferase Reporter Assay Kit (Cat#: FR201, TransGen, China).

### Co-immunoprecipitation experiment

The co-immunoprecipitation (Co-IP) experiment was conducted using Pierce Classic magnetic immunoprecipitation kit (Cat#:88804, Thermo Fisher Scientific, USA). Proteins were immunoprecipitated from cell lysates with primary antibodies (anti-NEDD4 antibody, anti-UB antibody, anti-PTEN antibody, and anti-IgG antibody are listed in Supplementary Table 3). Protein complexes were bound to Protein A/G Magnetic Beads at 25 °C for 1 h, the beads were washed twice with wash buffer and once with ddH_2_O, and the protein complexes were eluted with low-pH buffer and analyzed by western blots. The secondary antibody was replaced by HRP-conjugated anti-rabbit IgG, Light Chain Specific (SA00001-7L, Proteintech, Wuhan, China); the remaining steps are as described above.

### Immunofluorescence

K562 or K562/DOX cells (2 × 10^5^ cells/well) were inoculated into 24-well plates and transfected with 100 nM miR-92b-5p mimic or inhibitor for 6 h, followed by a 48-hour culture period. The cells were washed twice with PBS and resuspended in 0.1 mL PBS (2-5 × 10^6^ cells/mL). Shi-fix^TM^ cover-slips (#SB-Shifix25, Shikhar Biotech, Lalitpur, Nepal) were placed in a 12-well plate. The cell suspension was added directly onto the Shi-fix^TM^ cover-slips. The samples were incubated at room temperature for 30 min, after which unbound cells were gently removed by washing with 1 mL PBS. The Shi-fix^TM^ cover-slips were fixed, permeabilized, and then incubated with primary antibodies (bs-0748R, Bioss Biotechnology, listed in Supplementary Table 3) overnight at 4 °C and HRP-linked anti-rabbit IgG secondary antibody (AFIHC003, Aifang biological, Changsha, China) for 30 min at 25 °C in 12-well plate. Afterward, the Shi-fix^TM^ cover-slips were incubated with TYR-570 reagents (AFIHC027, Aifang biological, Changsha, China) and DAPI (AFIHC044, Aifang biological, Changsha, China) and visualized in Digital Pathology Scanner (KF-FL-020, KFBIO).

### CCK-8 assay

A total of 1 × 10^4^ cells were cultured with different concentrations of doxorubicin and for 48 h in 96-well plates. The CCK-8 solution (A311, Vazyme, Nanjing, China) was added; after incubation for 4 h, the OD value was measured at 450 nm wavelength using the multimode plate reader (EnVision Xcite, USA).

### Apoptosis detection by flow cytometry

A total of 5 × 10^5^ cells were cultured with 5 μM doxorubicin and for 4 h. After treatment, cells were collected and gently washed with cold PBS twice. Then, cells were resuspended in 500 μL 1× binding buffer, 5 μL Annexin V-FITC, and 5 μL PI using Annexin V-FITC/PI Apoptosis Detection Kit (KGA1102-50, KeyGen, Jiangsu, China). The stained cells were incubated in the dark for 5 min at room temperature and then analyzed using a flow cytometer (DxP Athena^TM^, Cytek Biosciences).

### Statistical methods

Triplicates of all experiments were performed at least. For the data in this study, all were presented in the form of mean ± standard deviation. Data comparison between two groups was performed using a *t*-test, while comparisons among multiple groups were conducted using one-way ANOVA. Statistical significance was set at *P* < 0.05. (^*^P < 0.05, ^**^P < 0.01, ^***^P < 0.001, ^****^P < 0.0001, *n* = 3).

## RESULTS

### Doxorubicin resistance-related exosomal miRNA in cell lines and AML patients

**Figure 1:**
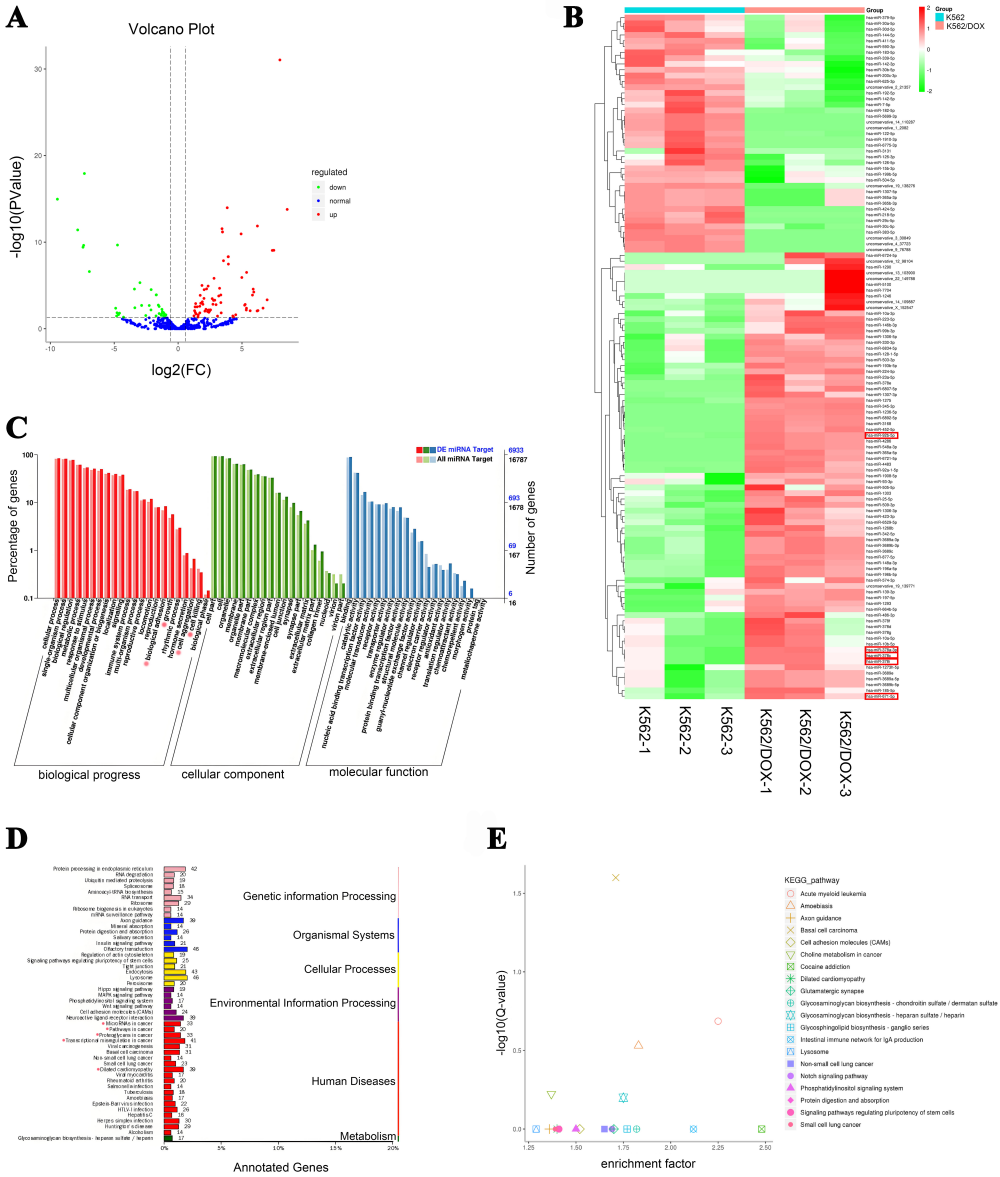
Comprehensive analysis of differentially expressed exosomal miRNAs between doxorubicin-resistant K562/DOX cells and the doxorubicin-sensitive K562 cells and their target genes. (A) Volcano plot of differentially expressed exosomal miRNAs; (B) Cluster map of differentially expressed exosomal miRNAs; (C) GO analysis of differentially expressed exosomal miRNAs target genes; (D) KEGG analysis of differentially expressed exosomal miRNAs target genes; (E) KEGG pathway enrichment of differentially expressed exosomal miRNAs target genes. miRNAs: MicroRNAs; GO: gene ontology; KEGG: Kyoto Encyclopedia of Genes and Genomes.

To explore the mechanisms underlying doxorubicin resistance transferred by exosomes, we investigated exosomal miRNA profiles in doxorubicin-resistant cells and chemoresistant AML patients through high-throughput miRNA sequencing. The results revealed 77 exosomal miRNAs were upregulated and 41 exosomal miRNAs were downregulated in the doxorubicin-resistant K562/DOX cells compared with doxorubicin-sensitive K562 cells [Figure 1A and B], 64 exosomal miRNAs were upregulated and 36 exosomal miRNAs were downregulated in chemoresistant AML patient [Supplementary Figure 2A and B]. The GO analysis suggested that the predicted target genes of differentially expressed exosomal miRNAs in doxorubicin-resistant K562/DOX cells may be associated with biological processes such as “cell aggregation”, cellular components like “extracellular matrix part collagen”, and molecular functions including “protein binding transcription factor activity”, “translation regulator activity”, and “chemorepellent activity” [Figure 1C]. To further elucidate the biological roles of these predicted target genes in doxorubicin-resistant K562/DOX cells, a KEGG analysis was conducted. This analysis identified key pathways potentially involving these target genes, with “Protein processing in endoplasmic reticulum”, “Olfactory transduction”, “Lysosome”, “Endocytosis”, and “Transcriptional misregulation in cancer” emerging as the top five [Figure 1D]. Furthermore, KEGG enrichment analysis indicated significant associations between the predicted target genes and pathways such as “Basal cell carcinoma”, “AML”, “Amoebiasis”, “Glycosaminoglycan biosynthesis - heparan sulfate/heparin”, and “Choline metabolism in cancer” [Figure 1E]. Notably, the “AML” pathway showed the highest enrichment factor, suggesting a considerable level of involvement. This result implies a potential link between the differential expression of exosomal miRNAs in K562/DOX cells and the development of AML.

### Exosomal miR-92b-5p is related to doxorubicin resistance in AML patients and cell lines

**Figure 2:**
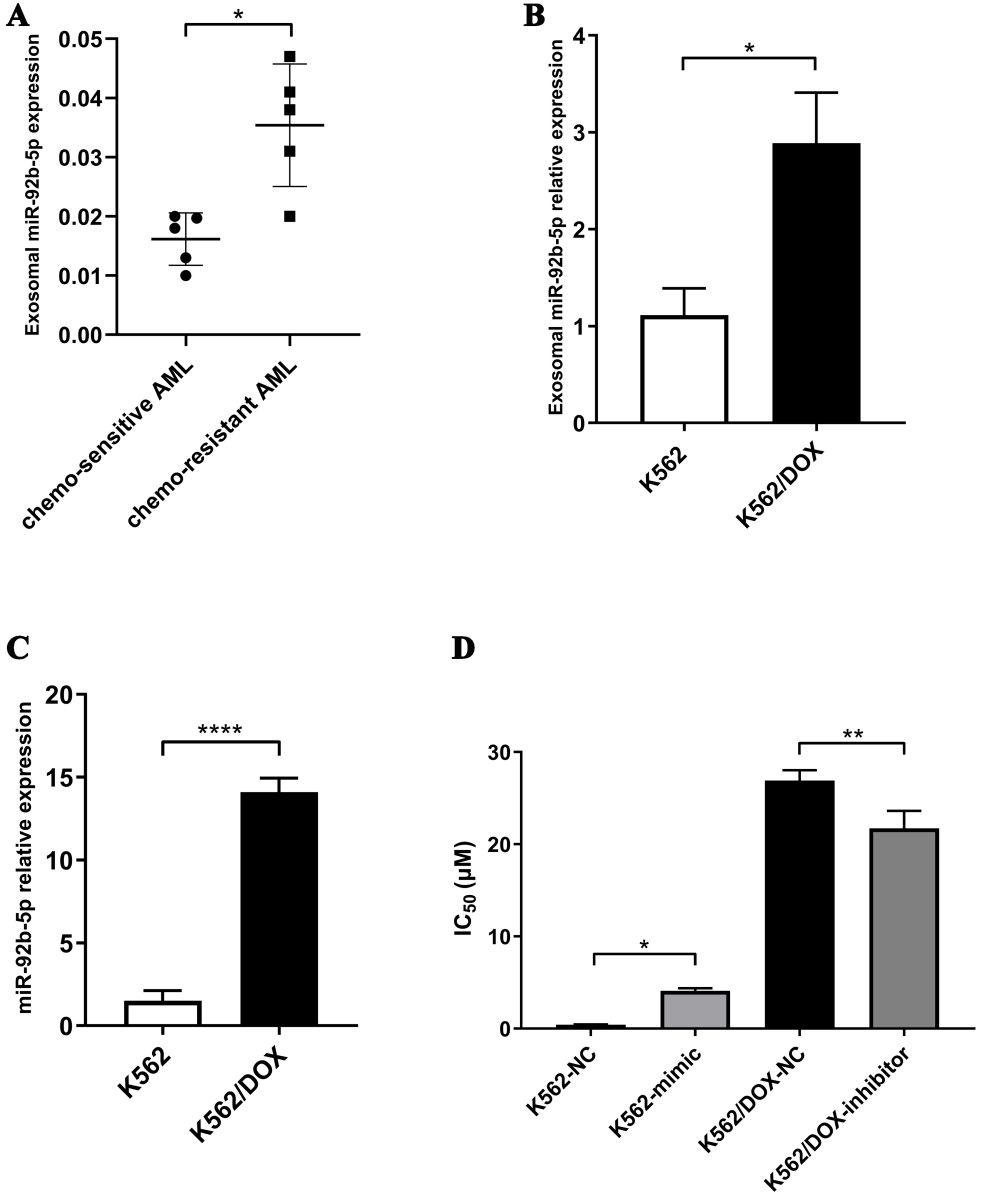
Exosomal miR-92b-5p is related to doxorubicin resistance in AML patients and cell lines. (A) The expression of exosomal miR-92b-5p in AML patients; (B) The expression of exosomal miR-92b-5p in K562 and K562/DOX cells; (C) The expression of miR-92b-5p in K562 and K562/DOX cells; (D) The IC_50_ value of doxorubicin in K562 and K562/DOX cells. ^*^P < 0.05, ^**^P < 0.01, ^****^P < 0.0001, *n* = 3. AML: Acute myeloid leukemia.

Based on exosomal miRNA-seq, we identified 10 miRNAs that exhibited increased expression levels in both doxorubicin-resistant K562/DOX cells and in chemoresistant AML patients, as detailed in Supplementary Table 4. We focused on the top five - miR-92b-5p, miR-671-5p, miR-378a-3p, miR-378c, and miR-378i - for validation. Firstly, we conducted quantitative PCR to measure the expression levels of above 5 miRNAs in exosomes derived from 5 chemotherapy-resistant AML patients and 5 chemotherapy-sensitive AML patients, as well as from K562/DOX and K562 cells. The results confirmed the significantly higher exosomal miR-92b-5p levels in chemoresistant AML patients (*P* < 0.0106, Figure 2A) and in K562/DOX cells (*P* < 0.017, Figure 2B). Exosomal miR-378a-3p and miR-378c also showed increased expression in chemoresistant AML patients and K562/DOX cells [Supplementary Figure 3A-D], while exosomal miR-671-5p and miR-378i elevated in K562/DOX cells but not in AML resistant patients [Supplementary Figure 3E-H]. The validation studies have identified exosomal miR-92b-5p as the most highly expressed miRNA in K562/DOX cells, with the most marked differences; therefore, we have selected exosomal miR-92b-5p as a potential biomarker for further investigation. Then, we discovered a significant upregulation of miR-92b-5p in K562/DOX cells compared to K562 cells. (*P* < 0.0001, Figure 2C). To verify the correlation between miR-92b-5p and doxorubicin resistance, we conducted transfections with miR-92b-5p mimics or inhibitors to regulate its expression within cells. Overexpression via miR-92b-5p mimics resulted in enhanced doxorubicin resistance in K562 cells (K562-mimic), evidenced by an increase in the IC_50_ for doxorubicin, whereas the inhibition of miR-92b-5p in K562/DOX cells (K562/DOX-inhibitor) corresponded with a decrease in doxorubicin resistance, as reflected by a reduced IC_50_ [Figure 2D]. Moreover, we found that ABCB1 and ABCG mRNA levels are elevated in K562/DOX cells, but miR-92b-5p does not affect their expression in K562 or K562/DOX cells [Supplementary Figure 4].

### miR-92b-5p binds to and suppresses N4BP1 expression in doxorubicin-resistant leukemia cells

**Figure 3:**
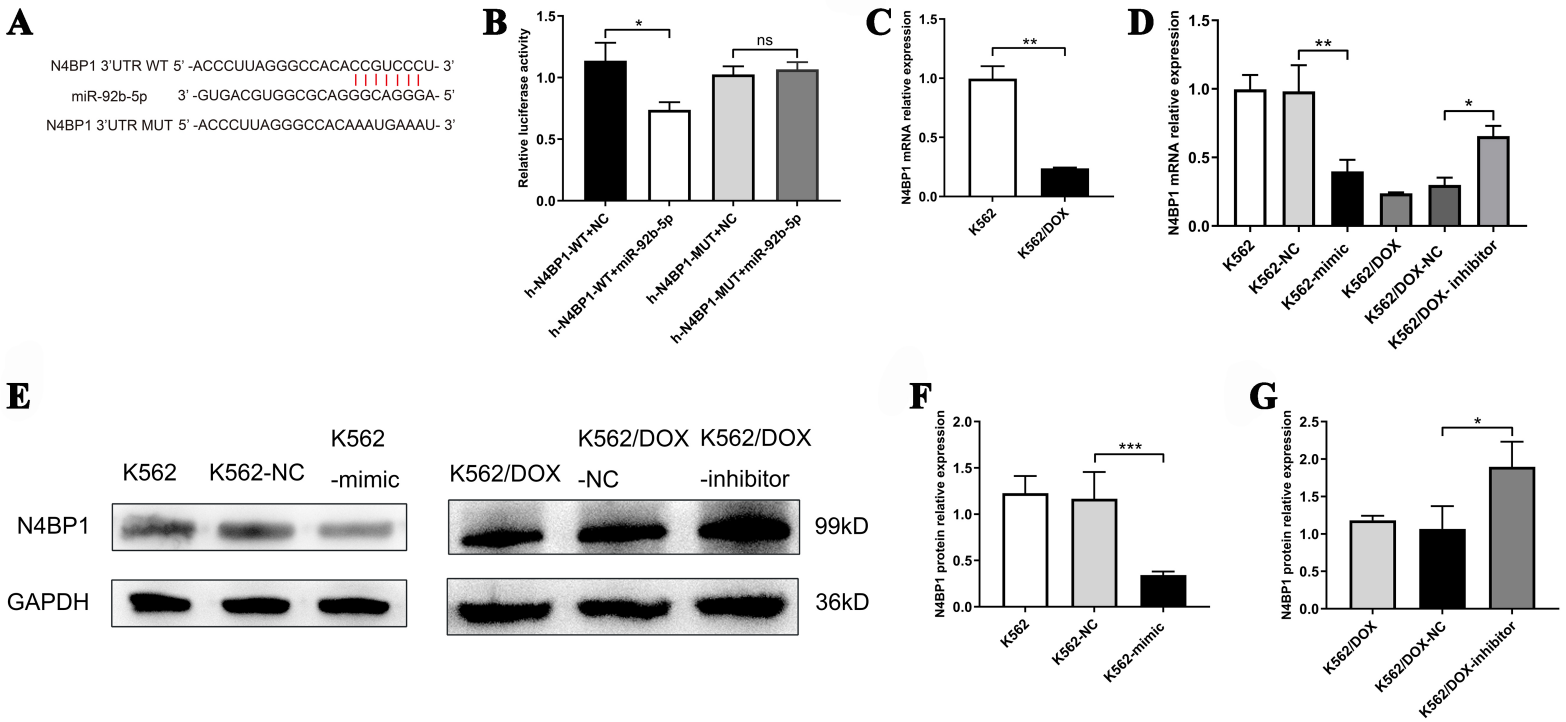
miR-92b-5p binds to and suppresses N4BP1 expression in doxorubicin-resistant leukemia cells. (A) Binding site of miR-92b-5p to N4BP1 mRNA 3’-UTR; (B) The relative Luciferase activity in 293T cells co-transfected with miR-92b-5p mimics or miR-NC and N4BP1-3’-UTR-WT or N4BP1-3’-UTR-MUT; (C) The expression of N4BP1 mRNA in K562 and K562/DOX cells; (D) The expression of N4BP1 mRNA in K562 and K562/DOX cells transfected with miR-92b-5p mimics or inhibitors; (E-G) The expression of N4BP1 protein in K562 and K562 /DOX cells transfected with miR-92b-5p mimics or inhibitors. ^*^P < 0.05, ^**^P < 0.01, ^***^P < 0.001, *n* = 3. N4BP1: NEDD4 binding protein 1.

N4BP1 was identified as a target gene for miR-92b-5p following bioinformatics analysis with TargetScan (https://www.targetscan.org, Figure 3A). This interaction was further validated by a dual-luciferase reporter gene assay, where co-transfection of miR-92b-5p mimics with the wild-type N4BP1 3’-UTR in 293T cells resulted in approximately a 38% decrease in relative luciferase activity, which was restored with mutated binding sites [Figure 3B]. Our q-PCR results showed that the N4BP1 mRNA was lower in doxorubicin-resistant K562/DOX cells compared with K562 cells (*P* < 0.01, Figure 3C), which is consistent with the high expression of miR-92b-5p in K562/DOX cells. Additionally, overexpression of miR-92b-5p in K562 cells diminished N4BP1 mRNA and protein levels, whereas its inhibition in K562/DOX cells increased N4BP1 expression, as illustrated in Figure 3D-G.

### NEDD4-mediated PTEN ubiquitination and its association with N4BP1 in doxorubicin-resistant leukemia cells

**Figure 4:**
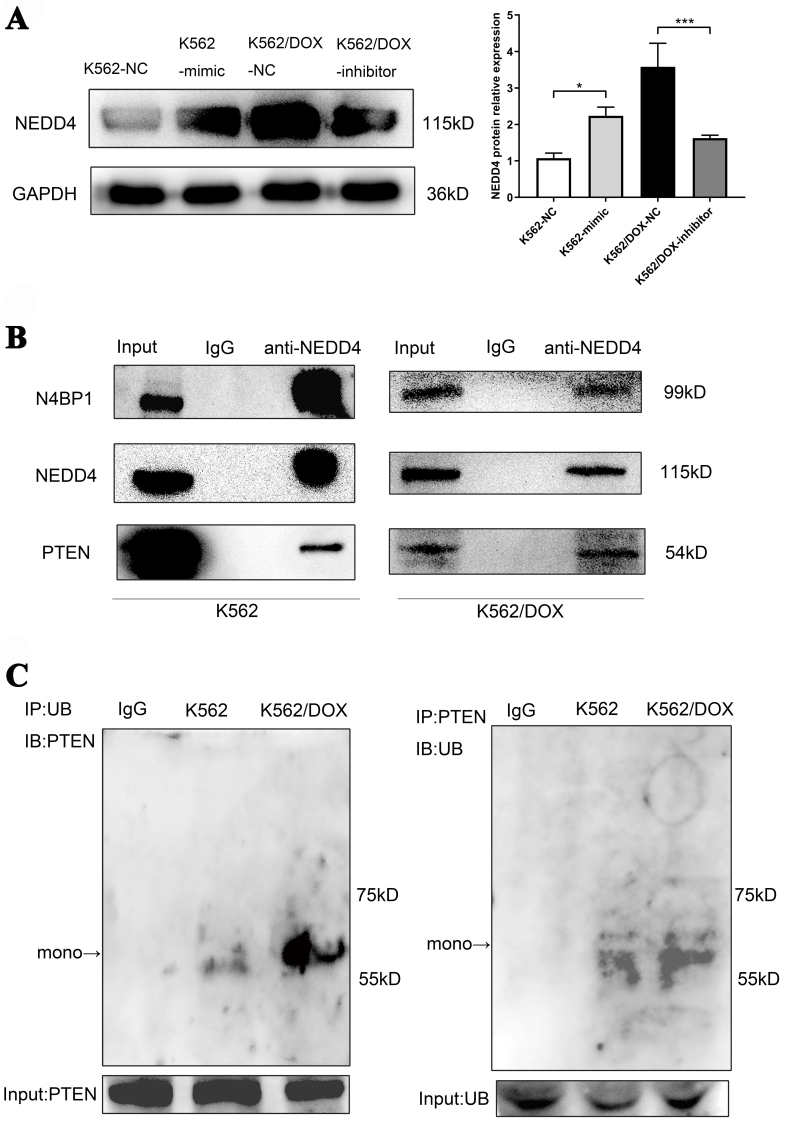
Analysis of NEDD4 expression, PTEN ubiquitination, and protein interactions in K562 and K562/DOX cells. (A) The expression of NEDD4 protein in K562 and K562/DOX cells transfected with miR-92b-5p mimics or inhibitors; (B) The expression of mono-ubiquitinated PTEN protein in K562 and K562/DOX cells; (C) NEDD4 and N4BP1, NEDD4 and PTEN endogenous Co-IP in K562 cells and K562/DOX cells. ^*^P < 0.05, ^***^P < 0.001, *n* = 3. N4BP1: NEDD4 binding protein 1; Co-IP: co-immunoprecipitation.

To confirm our hypothesis that N4BP1 participates in PTEN ubiquitination mediated by NEDD4, potentially affecting the doxorubicin resistance in leukemia cells, we assessed the expression of NEDD4 and the levels of PTEN ubiquitination in K562 and K562/DOX cells. Our findings indicate that overexpression of miR-92b-5p in K562 cells increased NEDD4 protein levels, whereas its inhibition in K562/DOX cells led to a decrease in NEDD4 expression [Figure 4A], which is consistent with the higher level of PTEN mono-ubiquitination assessed by Co-IP in K562/DOX cells as we expected [Figure 4B]. Furthermore, we performed Co-IP experiments using NEDD4 antibodies, and confirmed that NEDD4 is binding to N4BP1 and PTEN in both K562 and K562/DOX cells [Figure 4C].

### miR-92b-5p regulates RAD51 expression and PI3K-AKT-mTOR pathway via modulating PTEN subcellular localization

**Figure 5:**
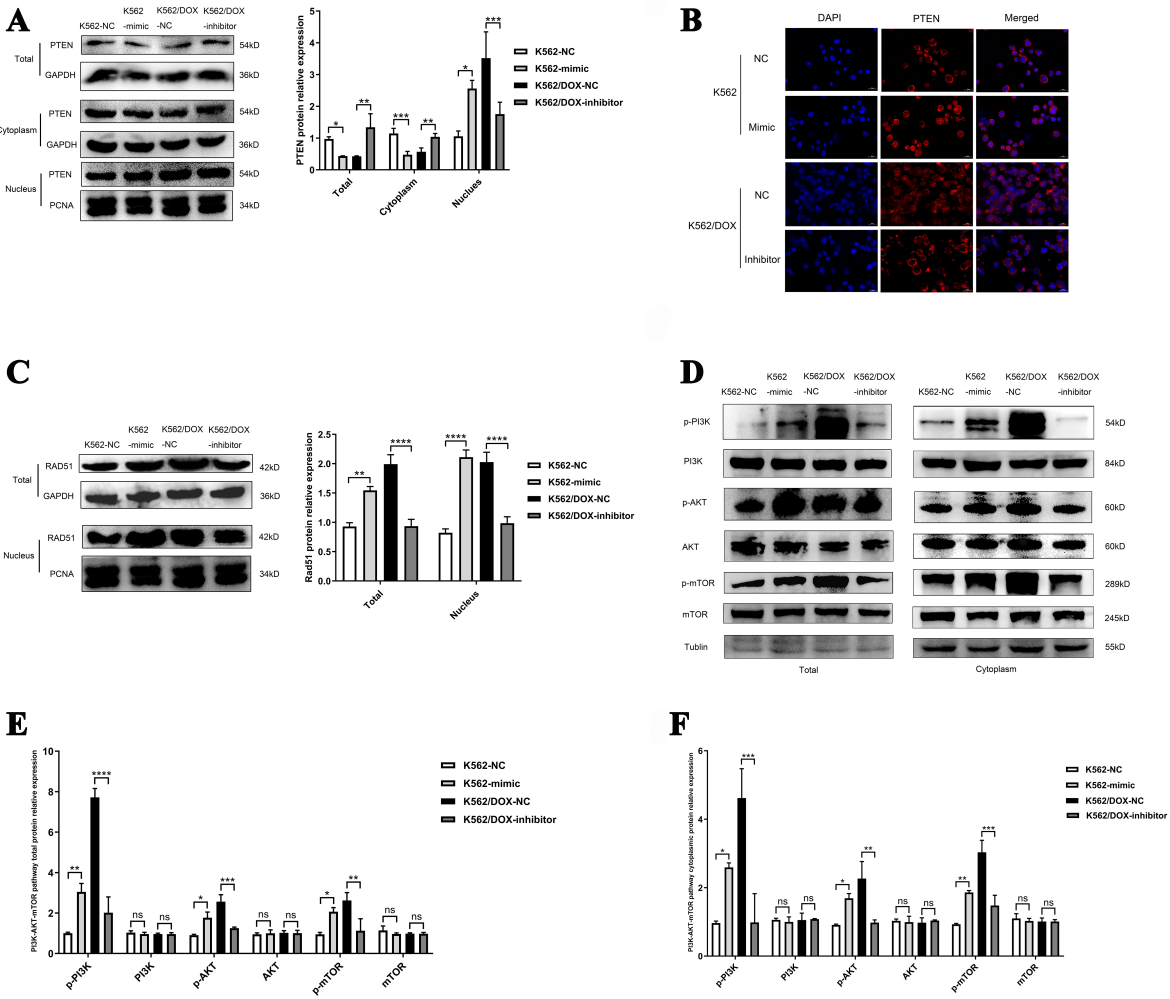
miR-92b-5p regulates RAD51 expression and PI3K-AKT-mTOR pathway via modulating PTEN subcellular localization. (A and B) The expression and relative expression of PTEN in total protein, cytoplasmic and nuclear proteins in K562 and K562/DOX cells transfected with miR-92b-5p mimics or inhibitors; (C) Immunofluorescence of PTEN in K562 and K562/DOX cells transfected with miR-92b-5p mimics or inhibitors; (D and E) The expression and relative expression of RAD51 in total protein and nuclear proteins in K562 and K562/DOX cells transfected with miR-92b-5p mimics or inhibitors; (F-H) The expression and relative expression of PI3K-AKT-mTOR pathway in total protein and cytoplasmic protein in K562 and K562/DOX cells transfected with miR-92b-5p mimics or inhibitors. ^*^P < 0.05, ^**^P < 0.01, ^***^P < 0.001, ^****^P < 0.0001, *n* = 3.

Given the influence of PTEN mono-ubiquitination on its nuclear distribution^[29]^, we subsequently explored the influence of miR-92b-5p on the subcellular expression levels of PTEN. Western blots results revealed that compared to the transfection of miRNA-mimic negative control (NC), transfection with miR-92b-5p-mimic in K562 cells increased nuclear PTEN protein, alongside decreased total and cytoplasmic PTEN protein. Consistently, compared to miR-inhibitor NC, miR-92b-5p inhibitor triggered a reduction in nuclear PTEN protein in K562/DOX cells, coupled with upregulated total and cytoplasmic PTEN protein [Figure 5A and B]. Immunofluorescence results further confirmed that miR-92b-5p modulates the subcellular localization of PTEN in both K562 and K562/DOX cells [Figure 5C]. Nuclear PTEN binds the RAD51 promoter, which is hypothesized to enhance homologous recombination repair^[30]^. Therefore, we investigated the influence of miR-92b-5p on RAD51 expression mediated by nuclear PTEN in leukemia. Our findings indicate that overexpression of miR-92b-5p in the K562 cells led to a notable rise in both total and nuclear RAD51 protein, while suppression of miR-92b-5p in K562/DOX cells caused a pronounced reduction in RAD51 levels in both compartments, aligning with our expectations. [Figure 5D and E]. On the other hand, we analyzed the PI3K-AKT-mTOR pathway to explore the function of cytoplasmic PTEN. We found that upregulation of miR-92b-5p elevated phosphorylated PI3K, AKT, and mTOR in both total and cytoplasmic proteins in K562 cells, while no substantial changes were observed in the expression levels of the PI3K, AKT, and mTOR in both total and cytoplasmic proteins simultaneously [Figure 5F-H]. These results highlight miR-92b-5p’s role in modulating PTEN localization, upregulating RAD51 expression and activating cell PI3K-AKT-mTOR pathway in leukemia cells.

### miR-92b-5p affects doxorubicin resistance through cell apoptosis and proliferation

**Figure 6:**
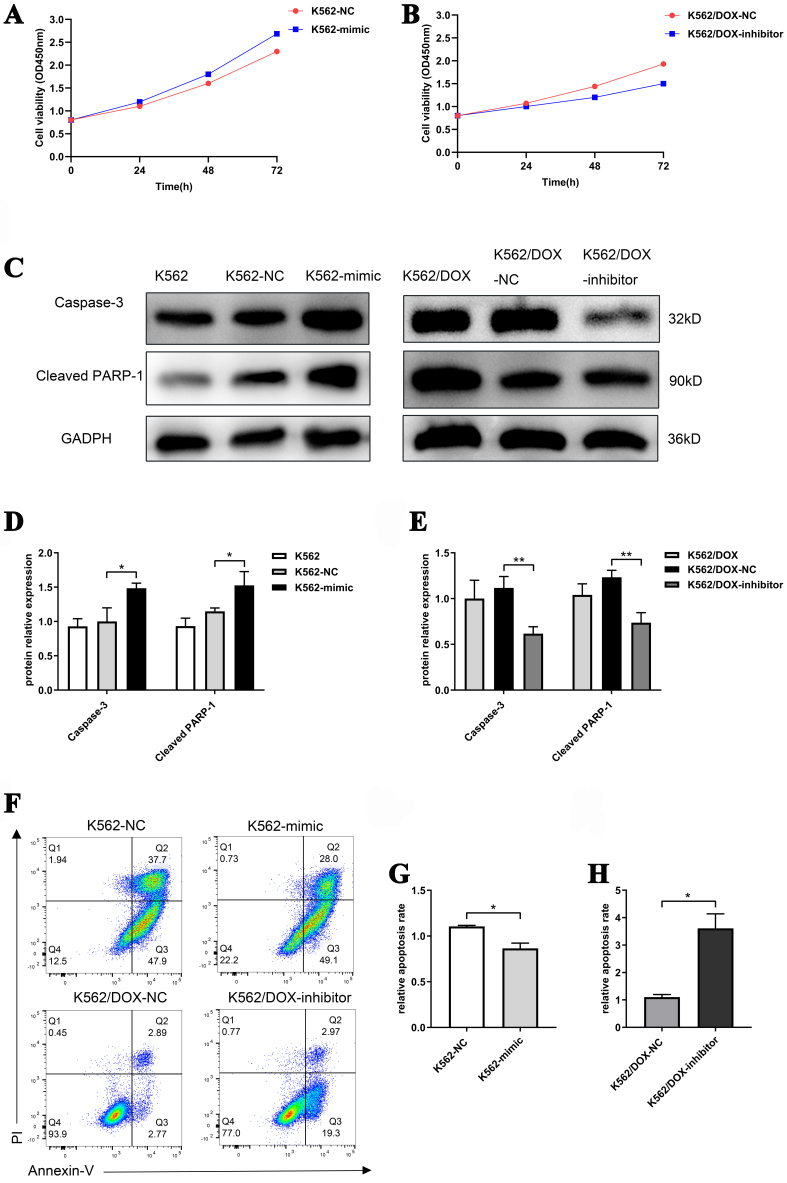
miR-92b-5p promotes cell proliferation and attenuates cell apoptosis. (A) The cell viability of K562 cells transfected with miR-92b-5p mimics; (B) The cell viability of K562/DOX cells transfected with miR-92b-5p inhibitors; (C-E) The expression and relative expression of capase-3 and cleaved-PARP1 proteins in K562 and K562/DOX cells transfected with miR-92b-5p mimics or inhibitors; (F-H). Apoptosis rate in K562 and K562/DOX cells transfected with miR-92b-5p mimics or inhibitors. ^*^P < 0.05, ^**^P < 0.01, *n* = 3.

Due to the activation of the PI3K-AKT-mTOR pathway promoting cell proliferation, and the enhancement of DNA damage repair by nuclear RAD51, thereby reducing cell apoptosis, we detected the effect of miR-92b-5p on the proliferation and apoptosis levels of K562 and K562/DOX cells. The CCK-8 assays demonstrated that the proliferation of K562 cells overexpressing miR-92b-5p (K562-mimic) was significantly higher than that of the control cells [Figure 6A]. Conversely, the proliferation of K562/DOX cells with miR-92b-5p suppression (K562/DOX-inhibitor) was notably reduced compared to the control cells [Figure 6B]. Western blot analysis revealed that the apoptosis proteins casepase-3 and cleaved-PARP1 in K562-mimic cells were reduced compared to the control cells [Figure 6C and D], whereas these proteins were elevated in K562/DOX-inhibitor cells [Figure 6C and E]. Flow cytometry corroborated these findings, showing that the apoptosis rate of K562-mimic cells was decreased compared to the control [Figure 6F and G], while the rate in K562/DOX-inhibitor cells was increased [Figure 6F and H]. Collectively, these findings substantiate the role of miR-92b-5p in augmenting cell proliferation and attenuating cell apoptosis.

## DISCUSSION

In this study, we conducted a comparative analysis of exosomal miRNAs between the parental K562 cells and the doxorubicin-resistant K562/DOX cells. MiRNAs-Seq analysis revealed a significant upregulation of 77 exosomal miRNAs in the K562/DOX cells, with miR-92b-5p exhibiting the most pronounced increase. Through GO and KEGG analyses, we identified that these miRNAs target genes involved in critical biological processes such as “biological adhesion”, “growth”, “cell aggregation”, “Transcriptional misregulation in cancer”, and “AML”. Further studies confirmed that miR-92b-5p directly targets N4BP1 mRNA, reducing its mRNA and protein levels. This downregulation of N4BP1 increases NEDD4 expression, promoting PTEN mono-ubiquitination and nuclear accumulation, while decreasing its total and cytoplasmic levels. Elevated nuclear PTEN induces RAD51 upregulation, enhancing DNA repair, while reduced cytoplasmic PTEN activates the PI3K-AKT-mTOR pathway, driving cell proliferation and inhibiting apoptosis. We propose that RAD51 and the PI3K-AKT-mTOR pathway are key downstream targets of miR-92b-5p in mediating doxorubicin resistance in leukemia.

The GO analysis revealed that the differential miRNA target genes in doxorubicin-resistant K562/DOX cells were enriched in “biological adhesion”, “growth”, “cell aggregation”, and were decreased in “cell killing”. An increase in “biological adhesion” and “cell aggregation” suggested the intercellular interactions and aggregation behaviors may increase in doxorubicin-resistant leukemia cells; this finding is consistent with that “biological adhesion” might correlate with doxorubicin resistance in leukemia^[31]^, and “cell aggregation” is thought to involved in drug resistance of AML cells ^[32]^. An increase in “growth” suggested that cell growth and proliferation might play a crucial role in doxorubicin-resistant leukemia. Moreover, a decrease in “cell killing” indicated that cell death and apoptosis in doxorubicin-resistant leukemia are diminished, which decreases the sensitivity of leukemia cells to cytotoxic stimuli, thereby promoting leukemia cell survival and inducing resistance to chemotherapy. In addition, KEGG analyses indicated these differential miRNAs target genes are enriched in “Transcriptional misregulation in cancer”, “MicroRNAs in cancer”, “Proteoglycans in cancer”, and “Pathways in cancer” in terms of “Human Diseases”. Moreover, KEGG pathway enrichment analyses identified “AML” as the most significant pathway, with the highest enrichment factor, which implied that these miRNAs could be involved in the development of cancer, especially in AML. It is worth mentioning that KEGG analyses indicated differential miRNAs target genes might also be involved in “Dilated cardimyopathy”, which suggests that these miRNAs may regulate cardiac muscle function and participate in the process of cardiac remodeling and functional deterioration. This result is consistent with that exosomal miR-92b-5p may serve as potential biomarkers for acute heart failure caused by dilated cardiomyopathy^[33]^.

Based on exosomal miRNA-seq, we identified 10 miRNAs that exhibited increased expression levels in both doxorubicin-resistant K562/DOX cell lines and in chemoresistant AML patients. Quantitative PCR validated that miR-92b-5p, miR-378a-3p, and miR-378c exhibited increased expression in K562/DOX cells and in resistant AML patients. Consistent with our findings, research revealed that chemotherapy triggers exosomal miR-378a-3p and miR-378d release via the EZH2/STAT3 axis, enhancing drug resistance by activating WNT/NOTCH pathways in breast cancer cells^[34]^. Our qPCR showed exosomal miR-671-5p and miR-378i elevated in K562/DOX cells but not in patients with treatment-resistant AML. The involvement of these miRNAs in leukemia remains understudied. In prostate cancer, miR-671-5p is known to drive tumor progression and metastasis by engaging the NFIA/CRYAB axis^[35]^. In colon cancer, miR-671-5p is associated with enhanced cell proliferation, migration, and invasion, correlating with a poorer prognosis^[36]^. Conversely, in glioblastoma multiforme, miR-671-5p modulates radiosensitivity via STAT3 and reduces tumor migration and cancer stem cell (CSC) characteristics by targeting TRAF2^[37]^. Given the differential expression patterns of exosomal miR-671-5p in AML patients, we speculate that the role of miR-671-5p may vary in different leukemia patients due to different genetic backgrounds. However, contrary to our findings, studies have found that miR-378i expression is reduced in tumor tissue of colon cancer patients^[38]^, and low concentrations of miR-378i may be associated with higher 5-year OS in liver cancer^[39]^. This discrepancy may stem from the distinct tumor types and their unique molecular landscapes. Notably, our findings reveal that three of the five most highly expressed genes are part of the miR-378 family, suggesting that this gene family may play a significant role in leukemia drug resistance mechanisms and warrants further investigation.

MiR-92b-5p is a member of the oncogenic miR-17~92 gene cluster, which is elevated in a variety of malignancies, including lymphomas, leukemias, lung cancers, breast cancers, and multiple myelomas^[40]^. Research has shown that miR-92b-5p is highly expressed in cholangiocarcinoma patients^[41]^ and cervical cancer patients with lymph node metastasis^[42]^. In gastric cancer, TCGA database analysis found that miR-92b-5p is an independent poor prognostic indicator for gastric cancer, and the prognostic model index “ImmiRSig” composed of miR-92b-5p and eight other miRNA molecules was found to be associated with sorafenib and paclitaxel resistance^[43]^. Exosomal miR-92b-5p may play different roles in different tumors. Consistent with our study, the expression of exosomal miR-92b-5p is found to be increased in breast cancer patients and is related to the stage^[44]^, but its expression level is decreased in lung cancer patients^[45]^. Given our findings that exosomal miR-92b-5p levels are elevated in leukemia patients with doxorubicin resistance and that its suppression can restore sensitivity to the drug, we propose that miR-92b-5p could serve as a potential molecular marker for predicting AML chemoresistance and a potential target for therapeutic intervention.

In our study, we identified N4BP1 as a target gene for miR-92b-5p for the first time, a discovery we validated through dual-luciferase reporter assays and transfection studies. N4BP1 is a developmentally expressed protein interactor and monoubiquitylation substrate of NEDD4^[46]^, which is mainly located in the nucleus^[47]^. Research has indicated that N4BP1 is associated with various miRNAs in cancers, such as miR-151 in prostate cancer, miR-28-5p in ovarian cancer, and miR-151a-5p in lung cancer, playing a role in inhibiting tumor progression^[48-50]^. However, the precise mechanisms underlying N4BP1’s function have not been extensively elucidated in AML. In non-small cell lung cancer cells, N4BP1 binds to ITCH’s WW domain, preventing the polyubiquitination of p73α and c-Jun, which may inhibit tumor growth and increase chemosensitivity through cell cycle arrest and apoptosis^[51]^. Additionally, it has been documented that NEDD4 interacts with PTEN through its C2 and HECT domains^[52]^. In our study, Co-IP experiments substantiated the binding of NEDD4 protein to both N4BP1 and PTEN proteins, findings that are in broad agreement with our experimental outcomes. These interactions suggest a complex regulatory network involving N4BP1, NEDD4, and PTEN, which may have significant implications for AML therapeutic strategies.

NEDD4 is a pivotal player in tumorigenesis, modulating the ubiquitination and degradation of key proteins involved in cell proliferation, apoptosis, cell cycle regulation, migration, invasion, epithelial-mesenchymal transition, CSCs, and drug resistance^[53]^. NEDD4-1 ubiquitinates PTEN at K13 and K289, triggering both mono- and polyubiquitination. Polyubiquitinated PTEN is earmarked for proteasomal degradation, diminishing cytoplasmic PTEN and potentially fostering tumor development, while monoubiquitinated PTEN relocates to the nucleus, increasing nuclear PTEN levels and reducing cytoplasmic levels^[29,54-56]^. Our findings indicate that PTEN is predominantly monoubiquitinated in doxorubicin-resistant leukemia cells, with a concomitant rise in nuclear PTEN. Moreover, studies have shown that the nuclear PTEN protein binds to the promoter region of RAD51, leading to radio-resistance by promoting homologous recombination repair in glioblastoma^[30]^ and temozolomide resistance^[57]^. The *RAD51* gene family is pivotal in the cellular response to double-strand DNA breaks, managing replication stress, and therefore maintaining genomic integrity and cellular survival. As an ATPase, RAD51 is instrumental in the search for and binding to homologous DNA sequences, thereby facilitating precise and efficient DNA repair^[58]^. Experiments have demonstrated that suppression of RAD51 expression leads to enhanced DNA damage and increased doxorubicin-induced apoptosis^[59,60]^. Consistent with these studies, we observed that the nuclear PTEN and RAD51 protein increased in K562/DOX cells and in K562 cells when miR-92b-5p was overexpressed. On the other hand, although we did not observe polyubiquitinated PTEN, our western blots results revealed a decrease in both total and cytoplasmic PTEN protein levels in K562/DOX cells and in K562 cells when miR-92b-5p was overexpressed. The decrease in cytoplasmic PTEN may result from the monoubiquitination of PTEN, which regulates PTEN’s translocation between the cytoplasm and the nucleus. Cytoplasmic PTEN, acting as a tumor suppressor, inhibits the PI3K-AKT-mTOR pathway through its phosphatase activity by dephosphorylating PIP3 to PIP2, which in turn suppresses cell proliferation and stimulates cell apoptosis^[61-63]^. These findings are in line with our experimental results that miR-92b-5p downregulates cytoplasmic PTEN protein and activates the PI3K-AKT-mTOR pathway in leukemia cell lines. Given our study’s results, we believe that RAD51 and the PI3K-AKT-mTOR pathway are key downstream effectors of miR-92b-5p that are implicated in mediating doxorubicin resistance in leukemia. However, the underlying mechanism for the reduction in total PTEN protein levels remains elusive. The exact pathway through which miR-92b-5p influences total PTEN levels warrants further investigation, including the exploration of post-transcriptional regulation and protein stability.

There are some limitations of this study. The clinical sample size in this study is small, which limits our ability to establish a robust correlation between exosomal miR-92b-5p expression levels and chemoresistance. To address this, we are committed to expanding our clinical sample collection to reduce the influence of random variability and to strengthen the validity of our findings. Additionally, our study focused on the K562 and K562/DOX cell lines, which, while informative, represent a narrow scope of the broader cellular landscape. In future research, we plan to develop a more diverse array of drug-resistant cell lines to provide a comprehensive understanding of the underlying mechanisms. Furthermore, our current research is confined to bioinformatics analysis and *in vitro* experiments, which, while valuable, do not fully capture the complexity of drug resistance in a clinical context. To bridge this gap, we will incorporate animal models in our future studies to explore the clinical relevance and therapeutic potential of our findings. Lastly, our study did not include the knockdown of NEDD4 and N4BP1, which are critical components in the PTEN mono-ubiquitination pathway. In subsequent studies, we will perform knockdown experiments to elucidate the roles of NEDD4 and N4BP1 in the context of PTEN regulation and doxorubicin resistance. This additional research will provide a more nuanced view of the molecular interactions and contribute to the development of targeted therapies for AML.

In conclusion, we consider that exosomal miR-92b-5p may be a potential molecular marker for predicting AML chemoresistance and a potential target for therapeutic intervention. Our study has demonstrated that miR-92b-5p plays a pivotal role in the regulation of PTEN mono-ubiquitination by suppressing the expression of N4BP1 mRNA and concurrently upregulating the expression of NEDD4 protein. This intricate molecular interplay leads to the nuclear translocation of PTEN, which in turn enhances DNA damage repair mechanisms by upregulating RAD51 and activates the PI3K-AKT-mTOR signaling pathway, thereby inducing doxorubicin resistance in AML. These discoveries shed new light on the resistance mechanisms in AML and offer a promising therapeutic target for clinical intervention.

## DECLARATIONS

### Availability of data and materials

The data used in this study were obtained from our laboratory experiments. The datasets generated and analyzed during the current study are available from the corresponding author upon reasonable request.

### Copyright

© The Author(s) 2025.
