# Supplementary material for: Exosomal miR-92b-5p regulates N4BP1 to enhance PTEN mono-ubiquitination in doxorubicin-resistant AML
Source: Cancer Drug Resist. 2025 Mar 28;8:16. doi: 10.20517/cdr.2024.140 (PMC11977356; doi:10.20517/cdr.2024.140)
Supplement: Supplementary file 1 [file cdr-8-16-SupplementaryMaterials.pdf]

Supplementary Materials

Exosomal miR-92b-5p regulates N4BP1 to enhance PTEN mono-ubiquitination in doxorubicin-resistant AML

Qianyuan Li[1,2], Jie Cheng[2], Danni Qin[2], Sheng Xiao[3], Chenjiao Yao[1,4]

[1]Department of General Medicine, The 3rd Xiangya Hospital, Central South University, Changsha 410013, Hunan, China.
[2]Department of Hematology, The 3rd Xiangya Hospital, Central South University, Changsha 410013, Hunan, China.
[3]Department of Pathology, The 3rd Xiangya Hospital, Central South University, Changsha 410013, Hunan, China.
[4]Department of Hematology, The First Affiliated Hospital of Hainan Medical University, Haikou 570105, Hainan, China.

Correspondence to: Prof. Chenjiao Yao, Department of General Medicine, The 3rd Xiangya Hospital, Central South University, 138 Tongzipo road, Yuelu District, Changsha 410013, Hunan, China. E-mail: yaochenjiao@csu.edu.cn

Supplementary Table 1. Clinical characteristics and diagnosis information in 10 AML patients

**Characteristic**	**sensitive AML**	**Chemo-resistant AML**
Diagnosis, *n*	M_{2b}, M_{2a}	M_{2a}, M_4, M_5
Male, *n*	2	2
Female, *n*	3	3
≥60y, *n*	1	1
<60y, *n*	4	4
Total, *n*	5	5

Supplementary Table 2. The primer sequences of the mRNAs and microRNAs

**Primer**	**Sequences**
miR-92b-5p-F	AGGGACGGGACGCGGTGCAGTG
miR-671-5p-F	AGGAGCCCTGGAGGGGCTGGAG
miR-378a-3p-F	CTCCTGACTCCAGGTCCTGT
miR-378c-F	GAGGCCATCACTGGACTTGG
miR-378i-F	ACTGGACTAGGAGTCAGAAGG
Universal-R	GCGAGCACAGAATTAATACGAC
PTEN-F	TTCTATGGGGAAGTAAGGAC
PTEN-R	ACAACAGTGCCACTGGTCT
N4BP1-F	TCAGGATACTTGTGCTGACCT
N4BP1-R	TGTGACTCCTAGCCATGACCA
U6-F	CTGCTTCGGCAGCACA
U6-R	TGGTGTCGTGGAGTCG
GAPDH-F	CCCATGGCAAATTCCATGGCACCG
GAPDH-R	GTCATGGATGACCTTGGCCAGGGG

Supplementary Table 3. The primary antibodies used for western blots

Anti-body	Host	Dilution	Catalog
anti-N4BP1	Rabbit	1:1000	R25077, Chengdu ZENBIO Biotechnology
anti-NEDD4	Rabbit	1:5000	R26203, Chengdu ZENBIO Biotechnology
anti-PTEN	Rabbit	1:1000	BS1305, Bioworld Technology
anti-RAD51	Rabbit	1:1000	BS3771, Bioworld Technology
anti- p-PI3K	Rabbit	1:1000	AF3242, Affinity Biosciences
anti- PI3K	Rabbit	1:1000	R22768, Chengdu ZENBIO Biotechnology
anti-p-AKT	Rabbit	1:1000	BS4007, Bioworld Technology
anti-AKT	Rabbit	1:1000	342529, Chengdu ZENBIO Biotechnology
anti-p-mTOR	Rabbit	1:1000	R25033, Chengdu ZENBIO Biotechnology
anti-mTOR	Rabbit	1:500	WL02477, Wanleibio
anti-caspase-3	Mouse	1:500	sc-7272, Santa Cruz Biotechnology
anti-cleaved-PARP1	Mouse	1:500	sc-56196, Santa Cruz Biotechnology
anti-PTEN	Rabbit	1:500	bs-0748R, Bioss Biotechnology
anti-lgG	Rabbit		30000-0-AP, Proteintech (2ug/1mg total protein lysate)
anti-Ub	Rabbit	1:1000	ET1609-21, HUABIO
anti-PCNA	Rabbit	1:2000	R25294, Chengdu ZENBIO Biotechnology
anti-Tublin	Rabbit	1:1000	ab185224, abcam
anti-GADPH	Rabbit	1:10000	AP0063, Bioworld Technology
CD63	Mouse	1:1000	sc-5275, Santa Cruz Biotechnology
calnexin	Rabbit	1:1000	10427-2-AP, Proteintech
HSP90	Mouse	1:1000	sc-69703, Santa Cruz Biotechnology
Alix	Mouse	1:1000	sc-53540, Santa Cruz Biotechnology

Supplementary Table 4. 10 miRNAs demonstrated elevated expression levels in both K562/ADR cell lines and chemo-resistant AML patient

Gene	LogFC	Pvalue
hsa-miR-6807-5p	5.349679731	0.004249311
hsa-miR-92b-5p	3.843686186	1.06E-14
hsa-miR-671-5p	3.549786591	1.40E-08
hsa-miR-378g	2.414859713	0.006756603
hsa-miR-378d	2.362731628	0.007194859
hsa-miR-378f	2.311130823	0.010429869
hsa-miR-378a-3p	1.893104628	0.000782991
hsa-miR-378c	1.770703909	0.001132104
hsa-miR-378i	1.770687935	0.001138425
hsa-miR-574-3p	1.574970308	0.025216649

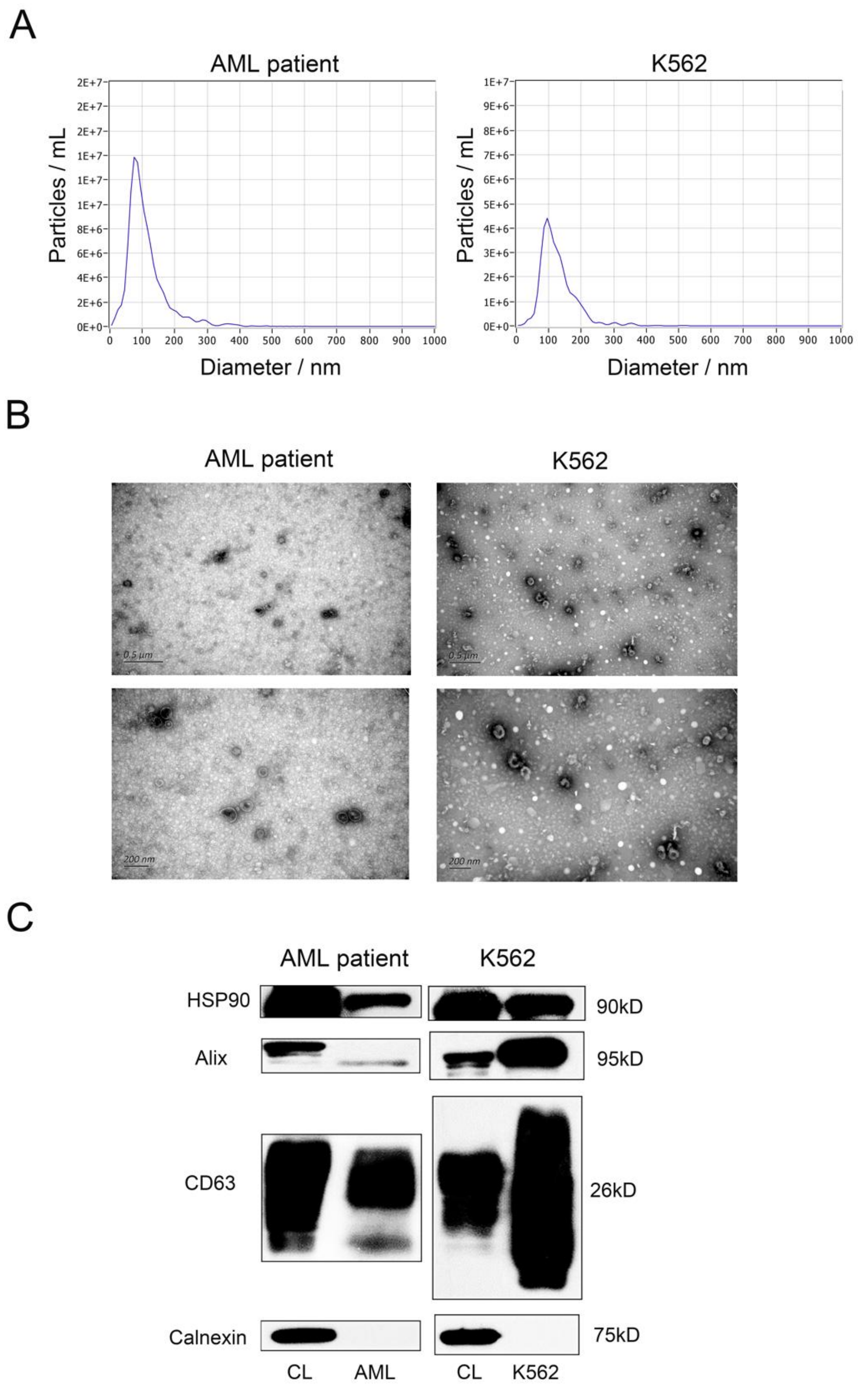

Supplementary Figure 1. The Identification of exosomes in K562 cells and AML patient. (A) Exosome nanoparticle size distribution; (B) The morphology of the exosome in Transmission Electron Microscopy; (C) The expression of calnexin, Hsp90, CD63 and Alix in exosomes.

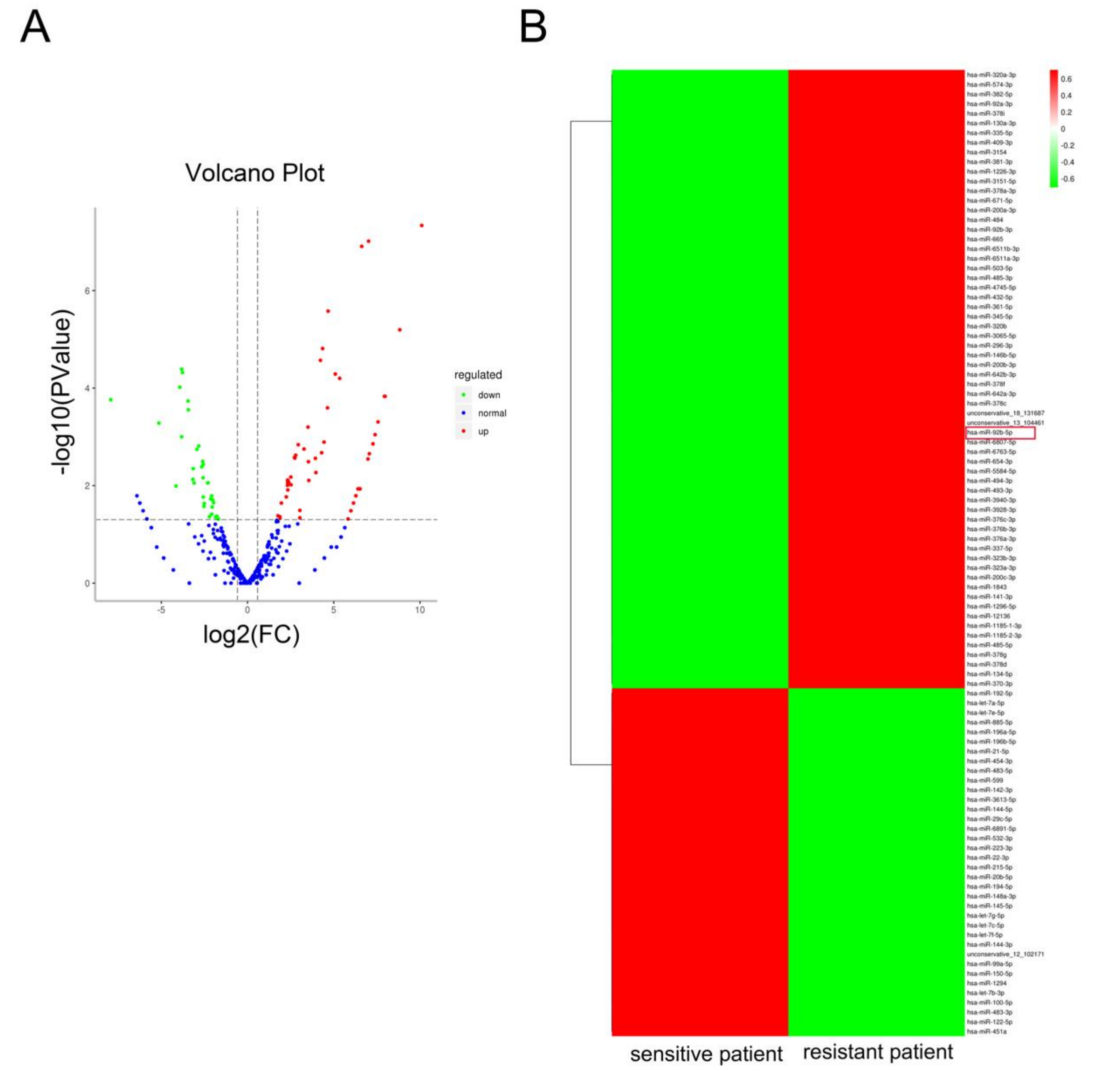

Supplementary Figure 2. Differentially expressed exosomal miRNAs in chemo-sensitive AML patient and chemo-resistant AML patient. (A) Volcano plot of differentially expressed exosomal miRNAs between chemo-sensitive AML patient and chemo-resistant AML patient; (B) Cluster map of differentially expressed exosomal miRNAs between chemo-sensitive AML patient and chemo-resistant AML patient.

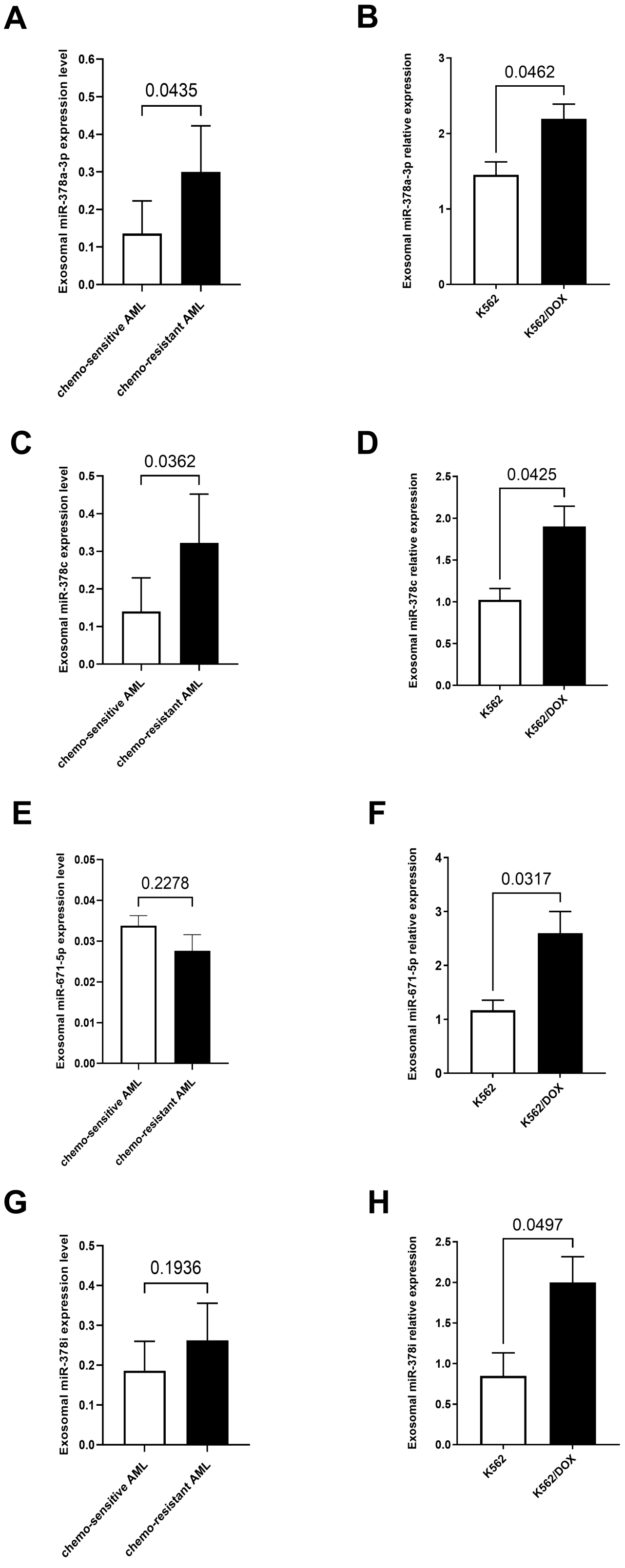

Supplementary Figure 3. Four exosomal miRNAs which highest expressed in chemo-sensitive AML patient and chemo-resistant AML patient, K562 and K562/DOX cells. Four candidate exosomal miRNAs expression in AML patients and cell lines (A) Exosomal miR-671-5p expression in 5 chemo-sensitive AML patients

and 5 chemo-resistant patients; (B) Exosomal miR-671-5p expression in K562 and K562/DOX cells; (C) Exosomal miR-378a-3p expression in 5 chemo-sensitive AML patients and 5 chemo-resistant patients. (D) Exosomal miR-378a-3p expression in K562 and K562/ DOX cells; (E) Exosomal miR-378c expression in 5 chemo-sensitive AML patients and 5 chemo-resistant patients; (F) Exosomal miR-378c expression in K562 and K562/ DOX cells; (G) Exosomal miR-378i expression in 5 chemo-sensitive AML patients and 5 chemo-resistant patients; (H) Exosomal miR-378i expression in K562 and K562/ DOX cells.

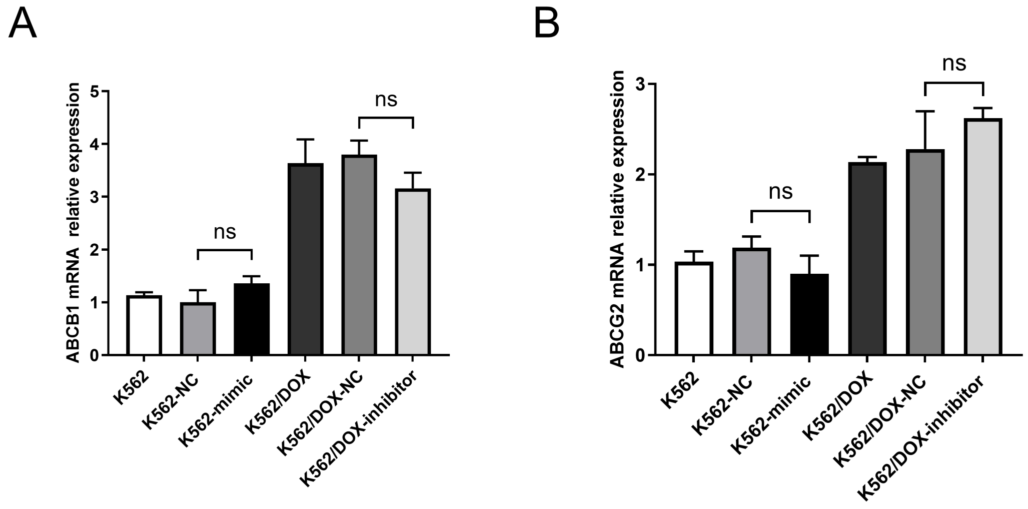

Supplementary Figure 4. The impact of miR-92b-5p on the expression of drug transport proteins (A) The expression of ABCB1 mRNA in K562 and K562/DOX cells transfected with miR-92b-5p mimics or inhibitors; (B) The expression of ABCG2 mRNA in K562 and K562/DOX cells transfected with miR-92b-5p mimics or inhibitors.
